# Novel influenza A antibodies reduce severity of secondary pneumococcal pneumonia after influenza infection in mice

**DOI:** 10.1186/cc14183

**Published:** 2015-03-16

**Authors:** KF Van der Sluijs, F Van Someren Greve, MD De Jong, MJ Schultz, NP Juffermans

**Affiliations:** 1 Academic Medical Center, Amsterdam, the Netherlands

## Introduction

Secondary bacterial pneumonia after influenza infection can cause severe disease with a high mortality. Recently, a new group 2 influenza A antibody (AT10_002) has been developed, which binds to multiple H3 and H7 subtypes. In a mouse model of primary influenza infection, treatment with AT10_002 as a fusion antibody protects against lethal infection, and reduces loss of bodyweight [1]. We hypothesized that treatment with AT10_002 reduces weight loss, lung injury and bacterial outgrowth, in a mouse model of viral infection followed by secondary pneumococcal infection.

## Methods

Male C57Bl/6 mice were intranasally inoculated with 400 TCID50 Influenza A (H3N2). Two days after infection, mice were injected with either AT10_002 i.v. (*n *= 8) or a control antibody (*n *= 7). After 7 days, both groups were intranasally inoculated with 5 × 10^3 ^*S. pneumoniae *type 3 and were sacrificed 18 hours later. Outcome measures were weight loss, wet lung weight, cell count in bronchoalveolar lavage fluid (BALF), and colony-forming units (CFUs) in lung homogenate. Data are represented as medians, and treatment groups are compared using nonparametric tests.

## Results

Mice receiving AT10_002 showed significantly lower weight loss at the time of sacrifice compared with the control group (+1% vs. -12% change in weight; *P *= 0.0003). Also wet lung weight was lower (68 vs. 96 mg; *P *= 0.0003), cell counts in BALF were lower (4.9 × 10^5^ vs. 7.0 × 10^5^ cells/ml; *P *= 0.0037) and CFUs in lung homogenate were lower (33 vs. 25 × 10^4^ CFUs/mg; *P *= 0.0003) compared with controls.

## Conclusion

Early treatment with influenza antibody AT10_002 significantly reduces weight loss, lung injury and bacterial outgrowth, in a mouse model of influenza infection followed by secondary pneumococcal pneumonia.

## References

[B1] Wagner K Proc Natl Acad Sci U S A 2014 111 16820 5 10.1073/pnas.1408605111 25385586 PMC4250106

